# Polysaccharide Regulation of Intestinal Flora: A Viable Approach to Maintaining Normal Cognitive Performance and Treating Depression

**DOI:** 10.3389/fmicb.2022.807076

**Published:** 2022-03-11

**Authors:** Xinzhou Wang, Lu Cheng, Yanan Liu, Ruilin Zhang, Zufang Wu, Peifang Weng, Peng Zhang, Xin Zhang

**Affiliations:** ^1^Department of Food Science and Engineering, Ningbo University, Ningbo, China; ^2^Department of Food Science, Rutgers, The State University of New Jersey, Newark, NJ, United States; ^3^Department of Student Affairs, Xinyang Normal University, Xinyang, China

**Keywords:** polysaccharides, intestinal flora, brain-gut axis, cognitive, depression

## Abstract

The intestinal tract of a healthy body is home to a large variety and number of microorganisms that will affect every aspect of the host’s life. In recent years, polysaccharides have been found to be an important factor affecting intestinal flora. Polysaccharides are widely found in nature and play a key role in the life activities of living organisms. In the intestinal tract of living organisms, polysaccharides have many important functions, such as preventing the imbalance of intestinal flora and maintaining the integrity of the intestinal barrier. Moreover, recent studies suggest that gut microbes can influence brain health through the brain-gut axis. Therefore, maintaining brain health through polysaccharide modulation of gut flora deserves further study. In this review, we outline the mechanisms by which polysaccharides maintain normal intestinal flora structure, as well as improving cognitive function in the brain *via* the brain-gut axis by virtue of the intestinal flora. We also highlight the important role that gut microbes play in the pathogenesis of depression and the potential for treating depression through the use of polysaccharides to modulate the intestinal flora.

## Introduction

So far, gut microbiota has been studied for decades. There is a large amount of experimental data confirming the key role of gut microbes in a living organism and calling the gut microbiome “the second genome of the human body” (Zhu et al., 2010), which shows the importance of intestinal flora is greatly recognized in academic circles. Nowadays, research on the association of intestinal flora with various diseases has developed rapidly, such as cancer, type 2 diabetes, and even neurodegenerative diseases (Liu et al., 2018; Sanchez-Alcoholado et al., 2020; Shi et al., 2020). Therefore, in addition to traditional treatment, the regulation of intestinal flora has become a new option for the treatment of related diseases. There are many factors that influence gut microbiology, such as age, antibiotic therapy, and diet (Rinninella et al., 2019), among which dietary factors occupy a significant position and have received the most widespread social attention. The influence of diet on intestinal flora is huge; different nutrients in different foods can promote the growth of some microorganisms in the intestine and inhibit the growth of others (Zmora et al., 2019). The study noted that the fecal microbiota of Italian children on a Western diet pattern consisted mainly of *Salmonella* and *Escherichia*. Still, the fecal microbiota of South American children on a traditional plant-based diet pattern consisted mainly of *Prevotella* enterotype (Conlon and Bird, 2014). In recent years, researchers have found that polysaccharides, either in food ingredients or artificially extracted from specific plants, play an important role in regulating intestinal flora and can provide effective treatment for related diseases.

Polysaccharides are widely found in nature, primarily in plants, animals, and microorganisms (Yang et al., 2020; Barnes et al., 2021; Cai et al., 2021; Murayama and Sugiura, 2021). Humans can obtain polysaccharides by ingesting the corresponding foods and obtain energy through a series of digestion and absorption, such as the starch in cereal foods, which is a critical source of energy (Lovegrove et al., 2017). After scientists’ unremitting research, the antioxidant, anti-aging and immunity-regulating functions of polysaccharides have been widely used (Tian et al., 2020; Ren et al., 2021). In addition to these well-known functions of polysaccharides, scientists have found that polysaccharides and intestinal microorganisms have an interactive relationship. Not all polysaccharides consumed from food can be digested and absorbed by the body. Some of the non-digestible but fermentable polysaccharides are used by intestinal microorganisms to produce various metabolites, such as short-chain fatty acids (SCFAs) (Ahmadi et al., 2017). SCFAs, as the main metabolites of intestinal microorganisms, occupy a critical position in intestinal physiology (Ahmadi et al., 2017; Dupraz et al., 2021). After several years of research, the role of SCFAs has not only been limited to regulating the body’s metabolism but also been found to play an essential role in gut-brain communication (van de Wouw et al., 2018; Silva et al., 2020).

With the progress and development of science, many experiments have demonstrated the interaction between gut microorganisms and the brain (Martin et al., 2018). In a study, the pathogenesis of irritable bowel syndrome (IBS) well demonstrated a bidirectional effect between the nervous system and the gut (Shaidullov et al., 2021). The central nervous system (CNS) in IBS patients might have abnormalities in processing signals from the intestine, and researchers found that a therapeutic effect could be achieved by regulating the patient’s intestinal flora (Mayer et al., 2014). In an experiment on germ-free (GF) mice, it was found that most chemicals in the blood of GF mice were synthesized by intestinal microorganisms and that these chemicals could affect the behavior and neuroendocrine response of GF mice (Galland, 2014). In addition, researchers have found that gut microbes contribute to brain development (Galland, 2014; Mohajeri et al., 2018). Therefore, regulation of intestinal flora becomes a viable new approach for the treatment of related brain disorders (Zhu et al., 2020). Typical diseases include cognitive dysfunction and depression, and scientists have likewise discovered a link between gut flora and these disorders and suggested the possibility of treating them by regulating gut flora (Galland, 2014; Mohajeri et al., 2018).

The mystery of using polysaccharides to regulate the gut microbiota to maintain normal cognitive function in the brain and improve depression has been gradually unraveled by scientists in recent years. This provides a new approach to treating depression and maintaining normal cognitive function. In this review, we review scientific findings on the regulation of gut microbial structure by polysaccharides, the utilization of polysaccharides by gut microbes, and the effects of gut microbes on the brain *via* the brain-gut axis. In addition, we will further explore the potential of modulating gut microbiota through polysaccharides to treat depression and maintain normal cognitive performance and provide an outlook on the development of this method in the future.

## Polysaccharides and the Effect of Polysaccharides on Intestinal Microbiota

### Polysaccharides

The structure of polysaccharides is relatively complex, and natural polysaccharides are composed of many monosaccharide residues, which are interconnected by oligosaccharide bonds (Cui, 2005; Verma et al., 2019). Although the structure of polysaccharides is very complex, with the development of science and technology, we have clarified the molecular structure of a part of polysaccharides and the related sugar units that make up polysaccharides (Cui, 2005). From the source, polysaccharides can be divided into plant polysaccharides, animal polysaccharides, and microbial polysaccharides (Verma et al., 2019). For instance, starch is a common polysaccharide in life, which is derived from rice, are polymerized from glucose (Li and Gilbert, 2018). Starch usually includes amylose and amylopectin, the difference between which is that amylose has a small number of long-chain branches and low molecular weights, while amylopectin has a large number of short-chain branches and high molecular weights (Li and Gilbert, 2018). Natural polysaccharides have the advantages of being non-toxic, stable, and easy to obtain, and are valued by various fields (Li and Gilbert, 2018). Nowadays, in addition to the functional foods we know, polysaccharides have a wide range of applications (Dai et al., 2021; Qi et al., 2021). With the further exploration of polysaccharide functions, polysaccharides have been used for treating related diseases, such as obesity and type 2 diabetes (Wu et al., 2019; Chen et al., 2020). In recent years, scientists have also discovered that polysaccharides have the ability to regulate the structure of intestinal flora and protect intestinal health (Ahmadi et al., 2017). These findings open a new door to the study of polysaccharides. Researches on polysaccharides are continuing, and more potential will be explored in the future.

### Utilization of Polysaccharides by the Intestinal Microbiota

**FIGURE 1:**
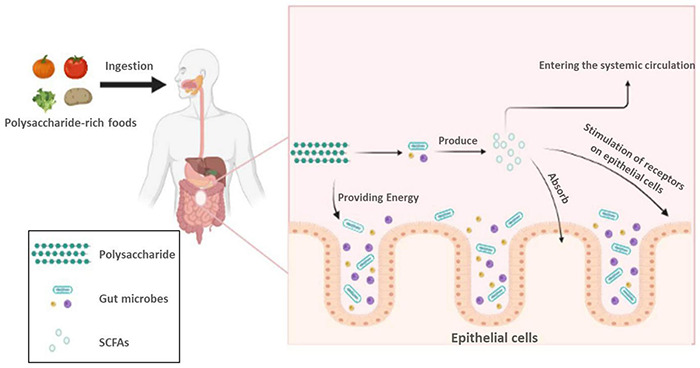
Utilization of polysaccharides by intestinal microorganisms.

Some polysaccharides obtained from the outside world cannot be digested and absorbed by the human body. These polysaccharides can be divided into two categories: fermentable and unfermentable. In contrast, unfermentable polysaccharides will eventually be discharged as waste, and fermentable polysaccharides can be degraded by intestinal microorganisms and eventually absorbed and used by the human body (Ahmadi et al., 2017). Previous research have pointed out some polysaccharides are required to be degradated by intestinal microorganisms for further utilization, such as resistant starch, levan, and mannan (Ye et al., 2020). Among the identified intestinal microorganisms, some of them are thought to be able to utilize polysaccharides, which they degrade by producing the corresponding enzymes (Bernalier-Donadille, 2010; Cockburn and Koropatkin, 2016), such as *Bacteroides*. *Bacteroides* occupy a large proportion of the intestinal microorganisms and are able to produce large amounts of carbohydrate-active enzymes (CAZymes) (Hemsworth et al., 2016; Ye et al., 2020). The researchers have found that CAZymes can degrade various polysaccharides and form fermentable monosaccharides, which facilitates the eventual absorption and utilization of polysaccharides by the body (Ye et al., 2020). Studies have demonstrated that certain intestinal microorganisms can provide themselves with a carbon source by fermenting polysaccharides, which will facilitate the production of important metabolites by intestinal microorganisms, such as SCFAs (Yao et al., 2020a). The utilization of polysaccharides by intestinal microorganisms is shown in Figure 1. SCFAs are important metabolites of intestinal microorganisms (Yao et al., 2020a); they are composed of a small hydrocarbon chain and a carboxylic acid moiety; the common SCFAs include butyric acid, acetic acid, propionic acid, etc. (Correa-Oliveira et al., 2016). SCFAs are relevant to our health. Studies have proven that SCAFs have weight loss and hypoglycemic effects, and can be used to treat obesity and type 2 diabetes (Murugesan et al., 2018; Sanna et al., 2019), as well as to regulate immune cell function and enhance immunity (Correa-Oliveira et al., 2016; Yao et al., 2020b). Moreover, in a recent study, researchers found that SCFAs also played an important role in the microbe-gut-brain axis (Dalile et al., 2019). The impact of SCFAs on microbial-gut-brain communication is dramatic (Dalile et al., 2019; Silva et al., 2020). In the past, a large number of studies have demonstrated that SCFAs can act on free fatty acid receptor 2 (FFAR2) and free fatty acid receptor 3 (FFAR3); among them, FFAR2 is mainly expressed in immune cells, and FFAR3 is mainly expressed in the sympathetic nervous system (Dalile et al., 2019). In addition, SCFAs also affect brain function by acting on the CNS, and based on this, researchers have found that SCFAs can relieve chronic psychological stress and have a therapeutic effect on related neurological disorders, such as depression and Parkinson’s disease (PD) (van de Wouw et al., 2018; Shin et al., 2020; Silva et al., 2020). With the continuous development of science and technology, the research on the utilization of polysaccharides by intestinal flora and the function of their metabolites will be further developed in the future.

### Shaping of Intestinal Flora by Polysaccharides

Among the many factors that influence the structure of the intestinal flora, diet is considered to be the easiest one to manipulate and most likely to reshape the intestinal flora. Polysaccharides, as part of the daily diet, also have a regulatory effect on the structure of the intestinal flora (Ho Do et al., 2021). The human body has to consume a certain amount of carbohydrates every day. Because the human body lacks the corresponding enzymes, some of the complex carbohydrates cannot be digested and absorbed by the body (Xu et al., 2013; Ho Do et al., 2021). As a representative of complex carbohydrates, part of polysaccharides can serve as an essential source of nutrients for intestinal microorganisms and influence the growth of related intestinal microorganisms, and thus regulating the structure of intestinal flora (Xu et al., 2013). In addition, polysaccharides also promotes the production of metabolites by intestinal microorganisms, which in turn changes the living environment of microorganisms, further shaping the structure of the intestinal flora (Xu et al., 2013). For example, Li et al. (2019) shown that the structure of the intestinal flora of rats could be significantly changed by adding a certain amount of *grifola frondosa* polysaccharides to the feed. In another experiment, Xu et al. (2017) used *Ganoderma lucidum* polysaccharides (GLP) to treat mice with intestinal flora disorders due to a high-fat diet and found that the treatment with GLP increased the abundance of *Enterococcus* in the intestines of the mice, which had a moderating effect on the intestinal flora structure of the mice. In addition, a large number of similar experiments have confirmed the shaping effect of polysaccharides on the structure of intestinal flora. For example, the study on coix polysaccharides found that it could promote the proliferation of *Lactobacillus* and *Bifidobacterium* in the intestine and increase the total amount of SCFAs, thus changing the structure of intestinal flora (Yin et al., 2018). In another experiment, researchers found by simulating gastrointestinal digestion and fermentation that *Pleurotus eryngii* polysaccharide (PEP) could not be digested by the gastrointestinal tract, but could be metabolized and utilized by intestinal microorganisms and produce the metabolites SCFAs, which also indicated that modulation of PEP polysaccharides could change the composition structure of intestinal microorganisms, as shown by a significant increase in the relative abundance of *Firmicutes* (Ma et al., 2021). Moreover, the ocean also contains many polysaccharides that we can study and use, and among them, seaweed polysaccharides have been studied most extensively. A study on brown seaweed showed that polysaccharides extracted from brown seaweed can be metabolized by intestinal microorganisms to produce SCFAs, and that the abundance of the phylum *Firmicutes* and *Bacteroidetes* was significantly increased in the intestine after brown seaweed polysaccharides modulation (Chen et al., 2018). Summarizing the above experimental results, we can easily conclude that polysaccharides that are not digested by the gastrointestinal tract but are fermentable can regulate the composition of the intestinal microorganisms. The role of polysaccharides in shaping the intestinal flora structure is still under investigation, and regulating the structure of the intestinal flora through the use of polysaccharides will be applied in an increasing number of fields in the future.

## Gut Microbiota’s Function: Maintaining Normal Cognitive Function Through the Brain-Gut Axis

### The Role of Intestinal Microbes in the Brain-Gut Axis

**FIGURE 2:**
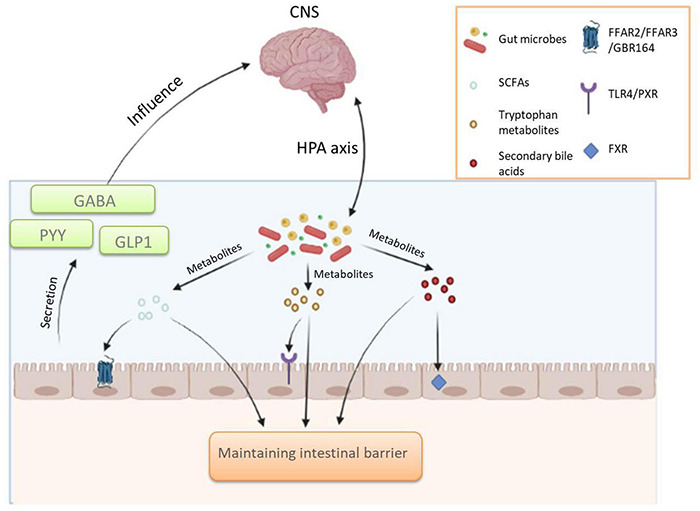
The role of gut microbial pairs in the brain-gut axis.

Studies have confirmed that there are at least three parallel channels between the gut microbes and the CNS that keep them in communication, involving endocrine, neural, and immune signaling mechanisms. According to the results obtained so far, it has been found that the gut microbes regulate the CNS mainly through neuroendocrine and neuroimmune mechanisms and that the vagus nerve plays an important role in this process (Martin et al., 2018). Communication between gut microbes and the CNS is usually mediated by molecules of microbial origin, including the SCFAs, tryptophan metabolites, and secondary bile acids, which mainly transmit signals to enterochromaffin cells (ECCs) and enteroendocrine cells (EECs). These molecules of microbial origin will induce the secretion of intestinal hormones, such as peptide YY (PYY), γ-aminobutyric acid (GABA), glucagon-like peptide 1 (GLP1), and transmits signals to the brain *via* the vagus nerve or body circulation (Martin et al., 2018; Dalile et al., 2019). A small percentage of molecules of microbial origin also enter the body circulation or cross the blood-brain barrier; however, it is not clear whether these molecules can reach and act directly on the brain (Martin et al., 2018; Dalile et al., 2019). In addition, it has been shown that intestinal microorganisms can indirectly affect the hypothalamic-pituitary-adrenal axis (HPA) through the blood circulating in the body. The HPA axis is closely related to some common psychiatric disorders (Dinan and Cryan, 2017). According to the available research results, the role of gut microbial pairs in the brain-gut axis is shown in Figure 2. To further discover the role played by gut microbes in the brain-gut axis, scientists have conducted numerous additional experiments. IBS has been shown to be caused by altered messages from the gut microbes to the brain, and the brain-gut axis plays a critical role in the pathogenesis of IBS (Mayer et al., 2014). One study found that some IBS cases were associated with changes in the metabolism and microorganisms that bound primary bile acids and proteases produced by intestinal microorganisms (Mayer et al., 2014). Moreover, extensive experimental evidence has shown that gut microbes can influence brain development and function through the brain-gut axis (Mayer et al., 2015; Rogers et al., 2016). One study in mice pointed out that altering the gut flora of mice during a certain developmental period affected brain development. In another experiment, functional brain responses were found to be altered in healthy women who consumed probiotics for a long period of time, as evidenced by their reduced response to an emotion recognition task (Mayer et al., 2015). In addition, multiple sclerosis (MS) is also affected by commensal microbes in the gut that act on brain cells, and by studying mouse models of the disease. The scientists found that metabolites from gut microbes altered the behavior of microglia in the brain, which in turn modulated astrocyte activity to promote or prevent inflammation (Rothhammer et al., 2018). The role of gut microbes in the brain-gut axis is undoubtedly enormous. Based on this, scientists are proposing more and more treatments for neurological diseases by regulating gut flora. Some of these methods have already been applied, and some need to be explored more.

### The Relationship Between Gut Microbes and Cognitive Performance and Improvement of Cognitive Performance Through Regulation of Gut Flora

As research continues to advance, it is increasingly recognized that intestinal flora plays an important role in the communication between the intestine and the brain. Therefore, there are more and more researches aiming at improving brain function by regulating intestinal flora. In recent years, it has been found that intestinal flora has a close connection with the cognitive ability of the brain, and regulating intestinal flora can play a certain role in improving cognitive ability (Davidson et al., 2018; Go et al., 2021; Kim et al., 2021). Cognitive abilities usually refer to the human brain’s ability to extract, store and process information, including imagination, memory, learning, and analytical ability (Lovden et al., 2020). Cognitive ability is a predictor of a society’s economic success, and the longevity and health of its members are an important part of the response to a society’s good or bad fortune (Lovden et al., 2020). Scientists have done a lot of research in the field of improving cognitive ability, and improving cognitive ability by regulating intestinal flora is a very promising direction. A large amount of experimental data suggests that the early life of an organism is an important period of gut microbial colonization and that affecting this period in some way will lead to changes in the structure of the organism’s gut flora, leading to certain effects on its cognitive abilities. For instance, the ability of bird to learn to sing will be affected by restricting the nutritional intake of birds, thus altering the composition structure of the microorganisms in their gut (Davidson et al., 2018). A growing number of experiments have demonstrated that regulating intestinal flora is effective in improving cognitive deterioration. The study of Ni et al. (2019) found that the use of probiotics, such as *Bifidobacterium* and *Lactobacillus*, had a significant effect on the regulation of intestinal flora and was effective in enhancing cognitive abilities, including learning and memory, in the elderly.

Diet is an important factor influencing gut flora, and there is a large amount of experimental data demonstrating that regulating gut flora through diet can have an impact on the cognitive ability of the brain (Zhang et al., 2020; Romanenko et al., 2021). In one experiment, researchers altered the intestinal flora of mice by changing their diet and found that mice fed a diet with beef had a significant improvement in memory, while another experiment showed that a diet high in sucrose led to impaired cognitive flexibility in mice (Leigh and Morris, 2020). In an experiment by Kang et al. (2014), they investigated the effects of exercise and a high-fat diet on the intestinal flora of mice and the effects of changes in intestinal flora on cognitive function, and the results showed that both exercise and a high-fat diet would have a significant effect on the composition of intestinal microorganisms, including the *Bacteroidetes* and *Firmicutes*, which are the most predominant in the intestine, and found that changes in intestinal flora through exercise could significantly improve cognitive decline due to dysbiosis caused by a high-fat diet. In addition, the researchers found that the regulation of intestinal flora by polysaccharides could greatly improve cognitive performance. Su et al. (2018) found that the composition of intestinal microorganisms in the mice treated with *Flammulina velutipes* polysaccharides (FVP) was significantly altered compared to the control group, as evidenced by an increase in the abundance of *Actinobacteria*, *Erysipelotrichia*, and *Bacteroidia*, and a decrease in the abundance of *Clostridia*, and that the FVP treated mice exhibited better learning and memory abilities. In another experiment, researchers found that *Cistanche deserticola* polysaccharides could inhibit oxidative stress and peripheral inflammation by restoring intestinal flora homeostasis due to D-galactose-induced aging in a mouse model, thereby improving cognitive function in mice (Gao et al., 2021). There are many other similar studies; for example, a polysaccharide extracted from Astragalus membranaceus has been shown to treat cognitive impairment by altering the intestinal flora of diabetic mice (Liu et al., 2019). The method of improving cognitive ability by regulating intestinal flora has received significant attention and will be applied in more and more fields.

As the role of intestinal flora in brain-gut communication becomes more apparent, researchers have found a strong link between intestinal flora and Alzheimer’s disease (AD) (Vogt et al., 2017). AD is a progressive neurodegenerative disease and a major cause of dementia. Patients with AD have significantly impaired cognitive abilities, which can manifest as memory loss and dramatic changes in daily behavior (MahmoudianDehkordi et al., 2019; Askarova et al., 2020). Until now, there has been no particularly good treatment for AD (MahmoudianDehkordi et al., 2019). This suggests that our understanding of AD still needs to be further developed. Based on the critical role that gut microbes play in the brain-gut axis, scientists are turning their attention to preventing and treating AD by regulating gut microbes (Xu and Wang, 2016; El Sayed et al., 2021). Studies have confirmed that AD pathology is characterized by extracellular plaques composed of amyloid-β peptides and intracellular neurofibrillary tangles composed of hyperphosphorylated tau protein. Based on the pathogenesis of AD, researchers have found that modulating gut microbes can affect brain amyloid deposition by studying AD mouse models (Jiang et al., 2017; Bostanciklioglu, 2019). In addition, the researchers found significant changes in intestinal flora diversity in AD patients, including decreases in the *Firmicutes* and *Bifidobacterium* and an increase in *Bacteroidetes* (Vogt et al., 2017). By using a comprehensive computational approach, the experiment demonstrated that the metabolites of intestinal microorganisms in humans might be an important mechanistic link between environmental exposure and various aspects of AD (Xu and Wang, 2016). In addition, experiments by MahmoudianDehkordi et al. (2019) similarly found a strong link between gut microbes and AD. It was found that serum concentrations of primary bile acids were lower in AD patients compared to normal elderly, while secondary bile acids, deoxycholic acid and its conjugated forms, produced by intestinal microbial metabolism, were higher. As the experiment progressed, the researchers found that the higher the levels of these secondary conjugated bile acids, the worse the cognitive function. Many experiments can give us some insight that the role played by intestinal microorganisms and their metabolites in the brain-gut axis have a certain influence on the pathogenesis of AD, and we can purposefully regulate the composition of the intestinal flora or influence the production of intestinal microbial metabolites to prevent the pathogenesis of AD, and the exploration of this field needs to be further advanced.

Given the important role of gut microorganisms in the brain-gut axis and the regulatory role of diet on gut flora, we can propose the hypothesis that the regulatory effect on gut flora can be achieved by changing the structure of the diet, such as increasing the intake of polysaccharides in the diet, and then improving cognitive performance and preventing some common diseases of cognitive decline through the effect of gut flora and its metabolites on the brain-gut axis. The regulation of gut flora as a new approach to improve cognitive performance has great scope for exploration in the future.

## Polysaccharides, Gut Microbiota, and Depression

### The Relationship Between Gut Microbiota and Depression

**FIGURE 3:**
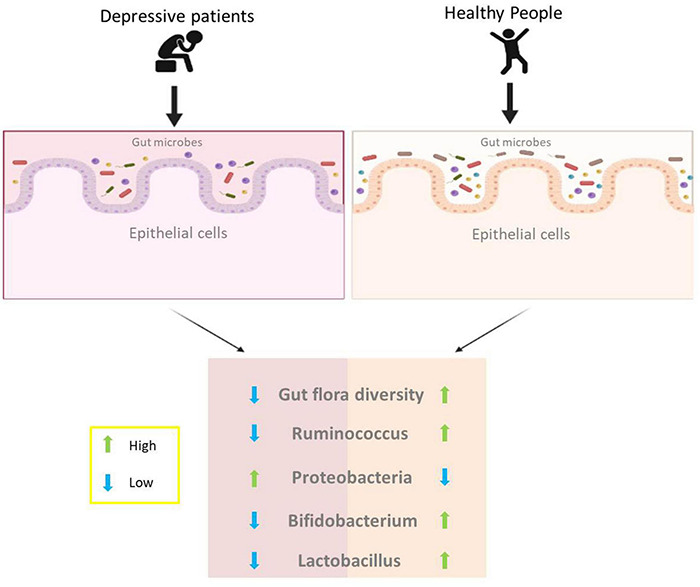
Differences in intestinal flora between depressed and healthy people.

Depression is one of the most common mental illnesses in everyday life and poses a serious health risk (LeMoult and Gotlib, 2019). Depression is often thought to be related to the regulation of neurotransmitters. To date, the causes of depression remain poorly understood, and multiple factors are involved in the development of depression (Malhi and Mann, 2018). Given the key role that gut microbes play in the brain-gut axis, scientists have identified a strong link between gut microbes and depression (Huang and Wu, 2021). According to the summary of a large amount of experimental data, the composition of gut microorganisms in depressed patients was significantly different compared to healthy controls in terms of the composition of gut microorganisms (Guo et al., 2019; Averina et al., 2020). The intestinal microbial diversity of depressed patients showed a significant decrease; more specifically, *Ruminococcus*, *Bifidobacterium*, *Faecalibacterium*, and *Lactobacillus* in the intestine of patients were at a lower level, and the level of *Proteobacteria* and *Bacteroides* was higher (Averina et al., 2020; Du et al., 2020), as illustrated in Figure 3. In general, there is a significant increase in harmful microorganisms and a decrease in beneficial microorganisms in the gut of depressed patients (Averina et al., 2020). The role played by gut microbes in the brain-gut axis has been mentioned above, and the metabolites produced by gut microbes can act as signaling substances to influence brain function. A study by Huang and Wu (2021) showed that gut microbes have a modulatory effect on brain neurotransmitters, and this modulation may have a role in the treatment of depression. In addition, in another experiment, researchers transplanted fecal flora from depressed patients into the intestines of GF mice and found that the mice showed depressive behavior, suggesting that changes in intestinal flora would lead to depression (Liu et al., 2020).

As we age, there is a certain aging of the intestinal flora in the human body, which is also associated with the development of depression (Linnemann and Lang, 2020). It was found that the core gut flora of older people showed some loss and weakness compared to younger people, such as differences in the abundance of *Bifidobacteria* (O’Toole and Jeffery, 2015; Kurilshikov et al., 2021). As mentioned above, gut flora can use dietary fiber in food to produce important metabolites such as SCFAs, which in turn affect the brain *via* the brain-gut axis. Thus, aging of the intestinal flora can impair the body’s ability to digest dietary fiber, resulting in malnutrition and neurodegeneration, leading to psychiatric disorders such as depression (Yahfoufi et al., 2020; Konjevod et al., 2021). From this, we can deduce that elderly people are more likely to suffer from depression due to the aging of intestinal flora, and can be prevented and treated by regulating the intestinal flora.

### The Interaction of Polysaccharides With Gut Microbiota to Improve Depression

The Health risks of depression are enormous, and a large number of depressed patients around the world participate in depression treatment every year, but the effectiveness of treatment is not obvious, and the withdrawal rate from treatment is very high (Kessler, 2018; Alexopoulos, 2019). The causes of depression are very complex, and according to studies to date, depression has been found to occur mainly in relation to life circumstances, mental state, physical health status, age stage, and genetics (Molenaar et al., 2016; Cepeda et al., 2019; Arias de la Torre et al., 2021; Dall’Aglio et al., 2021). Traditional treatments for depression include medication, electroconvulsive therapy, and some form of psychotherapy. Although these methods can provide some relief from depression, there are still many problems, such as the lack of effectiveness of the treatment for some patients and the unbearable treatment process (Holtzheimer and Nemeroff, 2006). Therefore, scientists hope to find a treatment that is effective and easy to use with few side effects (Cohen and DeRubeis, 2018).

With the continued exploration of gut flora, scientists have found a strong link between gut flora and the onset of depression and have produced some alleviating therapeutic effects on depressed patients by regulating their gut flora (Wu et al., 2020). Among the many factors affecting the structure of intestinal flora, diet is undoubtedly one of the most important factors, and different food components will have different effects on the intestinal flora, among which polysaccharides have received the attention of scientists with the advantages of easy access, little harm, and obvious regulation of intestinal flora (Van den Abbeele et al., 2020; Ho Do et al., 2021). Today, there are many experimental results showing that polysaccharides have the ability to reverse depression-induced dysbiosis of intestinal flora, anti-inflammatory, promote the production of SCFAs, and maintain the integrity of the intestinal barrier, which would contribute to the treatment of depression (Van den Abbeele et al., 2020; Shen et al., 2021; Yan et al., 2021). For instance, Yan et al. found high levels of pro-inflammatory cytokines in the hippocampus and serum of depressed mice and a significant dysregulation of intestinal flora in their experiments (Yan et al., 2020). By using polysaccharides extracted from okra, it was found that it had a significant restorative effect on the intestinal flora of depressed mice, as shown by an upregulation of the proportion of the *Firmicutes*, as well as a downregulation of the relative proportions of *Bacteroidetes* and *Actinobacteria*. This modulation would help to strengthen the intestinal mucosal barrier, as well as maintain normal intestinal immune system function and reduce the inflammatory response in the intestine, which is effective in combating depression, and depressed mice showed some improvement in their depressive symptoms. In addition, the experiment also revealed a significant increase in SCFAs in mice treated with okra polysaccharides, which act as important communication mediators and exert a positive influence on antidepressant disorders. In other experiments, similar results were obtained. Chen et al. (2019) found that a water-soluble polysaccharide from *Ginkgo biloba* effectively reversed depression-induced gut flora dysbiosis and also increased the abundance of *Lactobacillus*, thus providing a therapeutic effect on depression.

So far, the research on polysaccharide modulation of intestinal flora for depression is not deep enough, and this field still has very much room for exploration. Thinking further, this method of regulating intestinal flora using polysaccharides can effectively maintain the homeostasis of intestinal flora, prevent the loss and weakening of core intestinal flora, and is more convenient, safer and more acceptable than traditional depression treatments, offering a new possibility for the treatment of depression and allowing more depressed patients to receive reasonable treatment, especially elderly depressed patients. However, it is still too early to make this method widely available, and we need to conduct more in-depth research to ensure the effectiveness of this method. In the future, we can imagine that a special polysaccharide product may appear in our daily life, which can target the regulation of depression-related microorganisms in human intestine, improve the composition structure of intestinal flora, induce relevant intestinal microorganisms to produce beneficial metabolites, and help us prevent or treat depression, and in medical treatment, polysaccharide will also become an effective means to treat depression.

## Conclusion

Polysaccharides as a common food ingredient can effectively regulate the composition of microorganisms in the intestine and promote the production of metabolites from intestinal microorganisms. The metabolites of intestinal microorganisms can improve brain function through the brain-gut axis. According to recent experimental results, polysaccharides can effectively treat cognitive decline in the brain and improve depression by regulating intestinal flora, but the current research in this area is not thorough enough. In the future, the effects of polysaccharides on the regulation of gut bacteria and the production of gut microbial metabolites need to be further explored, which may provide a convenient, safe, and efficient treatment option for cognitive improvement and depression treatment.

## Author Contributions

XW: conceptualization, validation, and writing–original draft. LC: supervision. YL, ZW, and PW: writing–original draft. RZ: conceptualization. PZ: supervision and writing–original draft. XZ: supervision, writing–review, and editing. All authors contributed to the article and approved the submitted version.

## Conflict of Interest

The authors declare that the research was conducted in the absence of any commercial or financial relationships that could be construed as a potential conflict of interest.

## Publisher’s Note

All claims expressed in this article are solely those of the authors and do not necessarily represent those of their affiliated organizations, or those of the publisher, the editors and the reviewers. Any product that may be evaluated in this article, or claim that may be made by its manufacturer, is not guaranteed or endorsed by the publisher.

## Abbreviations

SCFAs: short-chain fatty acids
IBS: irritable bowel syndrome
CNS: central nervous system
GF: germ-free
CAZymes: carbohydrate-active enzymes
FFAR2: free fatty acid receptor 2
FFAR3: free fatty acid receptor 3
PD: Parkinson’s disease
GLP: *Ganoderma lucidum* polysaccharides
ECCs: enterochromaffin cells
EECs: enteroendocrine cells
PYY: peptide YY
GABA: γ-aminobutyric acid
GLP1: glucagon-like peptide 1
HPA: hypothalamic-pituitary-adrenal axis
FVP: *Flammulina velutipes* polysaccharides
AD: Alzheimer’s disease.
